# D-Fructose Exposure Impairs Neuronal Development in Mouse Neural Stem Cells

**DOI:** 10.3390/ijms27156653

**Published:** 2026-07-25

**Authors:** Jacqueline C. Hernandez, Mayara da Nóbrega Baqueiro, Lihiri Bora, Riya Singh, Marcio Alberto Torsoni, Adriana Souza Torsoni, Michael G. Ross, Mina Desai

**Affiliations:** 1 The Lundquist Institute, UCLA Medical Center, Torrance, CA 90502, USA; jacqueline.hernandez@lundquist.org (J.C.H.); lihiribora@gmail.com (L.B.); risingh178@gmail.com (R.S.); mikeross@ucla.edu (M.G.R.); 2 Laboratory of Metabolic Disorders, School of Applied Sciences, University of Campinas, Limeira 13484-350, SP, Brazil; mayarabaqueiro2009@hotmail.com (M.d.N.B.); torsoni@unicamp.br (M.A.T.); atorsoni@unicamp.br (A.S.T.); 3 Obesity and Comorbidities Research Center, University of Campinas, Limeira 13484-350, SP, Brazil; 4 Department of Obstetrics and Gynecology, David Geffen School of Medicine, University of California, Los Angeles, CA 90502, USA

**Keywords:** neural stem cell differentiation, neurogenesis, gliogenesis, neural morphology, soma, neurite length

## Abstract

Maternal obesity and a Western diet high in fat and sugars are increasing worldwide and may contribute to rising neurodevelopmental and neurobehavioral disorders in offspring. Both human and animal studies link these factors to adverse outcomes. However, the cellular mechanisms driving altered early-life neurogenesis, particularly in the hippocampus, remain unclear. To assess fructose effects on neural stem cell (NSC) differentiation and neuronal morphology, hippocampal NSCs from E12.5 C57BL/6 mouse embryos were cultured and treated with fructose (8.75 or 12.5 mM) for 7 days. Neuronal and astrocyte populations, along with neuronal morphology, were analyzed by immunofluorescence, and protein expression was assessed by Western blot. Fructose treatment significantly reduced neuronal and increased astrocyte counts, leading to a decreased neuron-to-astrocyte ratio compared to controls. These findings were supported by decreased MAP2 (neuronal) and increased GFAP (astrocyte) protein expression. Furthermore, fructose also significantly reduced neurite lengths without affecting neurite number. Morphological analysis revealed decreased soma size, reduced area/perimeter, and altered soma area-to-perimeter ratios, indicating impaired structural integrity. Thus, fructose exposure shifts NSC differentiation toward an astroglial lineage while suppressing neuronal development and impairs neuronal growth and structural complexity. Future studies are necessary to determine the influence of these cellular changes on synaptic plasticity and learning, as well as memory.

## 1. Introduction

There is a rising trend of neurodevelopmental disabilities among children in the United States [1]. This is consistent with recent studies demonstrating a high prevalence of attention deficit/hyperactivity disorder (9.6%), learning disability (7.5%), autism spectrum disorder (2.9%), intellectual disability (1.7%), and other developmental delays (5.2%) [1,2]. There have been numerous theories expounded for this high prevalence, including genetic predisposition, increased awareness and diagnosis [1], exposure to toxins, vaccines, infection, stress, poor nutrition, and others [3]. Although less well recognized, both human epidemiologic and animal model studies demonstrate that early-life exposure to maternal obesity and a Western diet is associated with adverse neurodevelopmental outcomes in offspring that extend to brain regions regulating neurobehavior [4].

The increase in maternal obesity (currently 34%) [5,6] has coincided with the marked rise in the rate of childhood neurodevelopmental disorders within the United States [1,7]. Human behavioral studies indicate that maternal obesity is associated with a 1.3- to 3.6-fold increase in the risk of intellectual disability or cognitive impairment in offspring [8]. The influence of Western diets, high in fructose/sucrose, refined carbohydrates, saturated fats, and low dietary fiber [9], has been shown to have a significant effect on the onset of maternal obesity [10] and neurodevelopmental disabilities in utero [11,12]. Furthermore, animal studies indicate that a maternal high-fat diet can negatively impair fetal neurodevelopment, resulting in decreased hippocampal neuronal cell counts, structural abnormalities, activation of fetal inflammatory cytokines, and the development of neuropsychiatric disorders (e.g., attention deficit hyperactivity disorder, autism spectrum disorder, anxiety, depression, schizophrenia) [13]. This is of concern, especially since fructose consumption in the US has risen dramatically over the past few decades from 16–20 g/day to 60–150 g/day due to the widespread use of fructose-containing sweeteners in processed foods and beverages [14,15]. Specifically, children and adolescents (aged 2–19) consume an average of approximately 68–81 g of added sugar daily, an amount that is nearly triple the recommended limit of 25 g per day for children over age two [16].

Among the brain regions, cognitive function is regulated within a complex system, including the prefrontal cortex, with the hippocampus playing a critical role [17]. Consistent with fetal brain development, the hippocampus begins developing during early embryonic life, with NPC proliferation, differentiation, and migration (neurogenesis) occurring throughout fetal life and after birth [18]. Postnatally, the hippocampus continues protracted functional and structural development. Specifically, the development of dendrites/synapses (crucial for functional maturation) [19,20], which is essential for the emergence of spatial learning and memory functions, is evident by the end of the third postnatal week in mice [21]. This postnatal period involves significant changes in neuronal connectivity and maturation [21].

Each of these periods plays a crucial role in the development of the nervous system, consequently making it vulnerable to any maternal environmental or nutritional insult [22]. Despite a myriad of research emphasizing detrimental abnormalities as a consequence of the maternal environment, there is limited knowledge of the effects of fructose during embryonic neurogenesis [23]. To elucidate this, we investigated the effects of in vitro fructose on NSC differentiation towards neuronal versus astroglial lineage and characterized morphological changes in neurons [24]. Determining the effects of fructose during early development provides an understanding of the observed childhood abnormalities and identifies potential treatment/preventative strategies.

## 2. Results

### 2.1. Fructose Suppresses Neurogenesis and Promotes Astrogenesis

**Figure 1:**
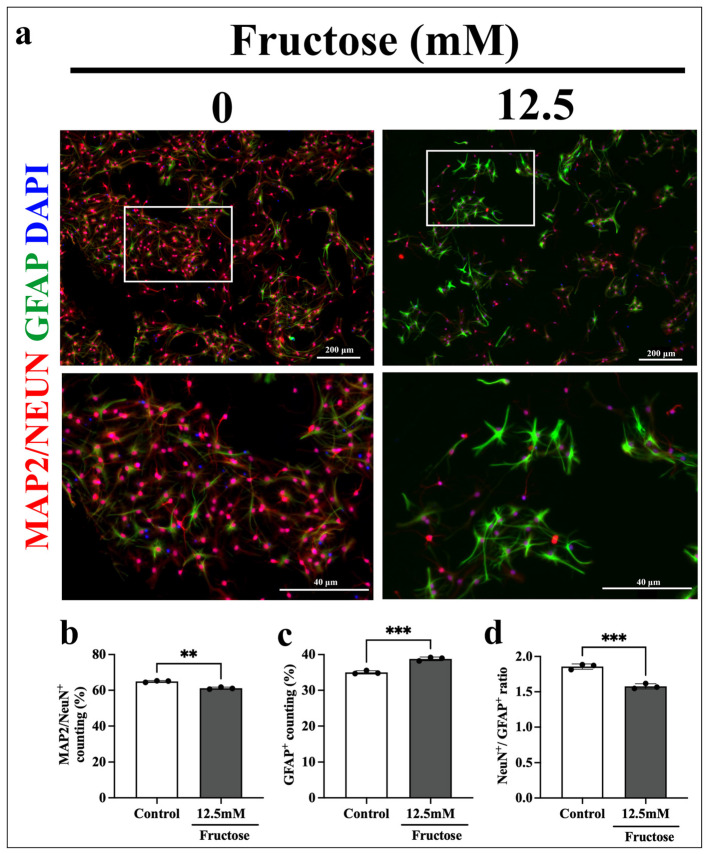
Effect of fructose on neuronal and astrocyte populations. NSCs were treated with 12.5 mM of fructose in differentiation medium for seven days. (**a**) Immunostained images at 10× magnification (top panel, scale bar = 200 μm) depicting neurons (MAP2/NeuN, red), astrocytes (GFAP, green), and nuclei (DAPI, blue), with corresponding zoomed-in white framed regions (lower panel, scale bar = 40 μm). (**b**) Neuronal counts as assessed by NeuN-positive cells. (**c**) Astrocyte count as assessed by GFAP-positive cells, and (**d**) ratio of neuron/astrocyte. An unpaired *t*-test was used to compare fructose-treated cells to controls. Values are mean ± SEM of *n* = 3 independent experiments. ** *p* = 0.0012, *** *p* = 0.0008.

In response to 12.5 mM fructose, a decreased number of neurons and increased astrocytes were visually evident in immunostained images (Figure 1a). This was confirmed by quantitative counting wherein NeuN/MAP2-positive cells showed a significant reduction as compared to untreated control cells (61.2 ± 0.3% vs. 64.9 ± 0.3%, *p* = 0.0012; Figure 1b). Conversely, GFAP-positive cells showed a significant increase in fructose treatments (38.8 ± 0.3% vs. 35.0 ± 0.3%, *p* = 0.0008; Figure 1c). Thus, the neuronal-to-astrocyte ratio decreased in response to fructose (1.6 ± 0.02 vs. 1.9 ± 0.02, *p* = 0.0008; Figure 1d).

**Figure 2:**
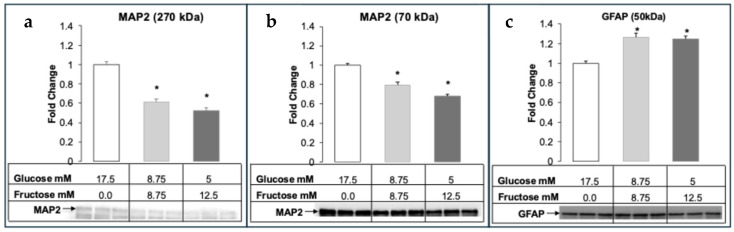
Effect of fructose on protein expression of neuronal and astrocyte markers. NSCs were treated with two doses (8.75, 12.5 mM) of fructose in differentiation medium for seven days. Protein expression of (**a**) MAP2 (270 kDa), (**b**) MAP2 (70 kDa), and (**c**) GFAP (50 kDa). Protein values are normalized to untreated control cells and expressed as fold change. Values are mean ± SEM of *n* = 3 independent experiments. Groups (Control □; 8.75 mM ■ and 12.5 mM ■ fructose) were compared using ANOVA with Bonferroni post hoc analysis. * *p* = 0.01.

Consistent with the cell count data, the protein expression of neuronal marker MAP2 (270 kDa), which is expressed during embryonic and postnatal periods, showed a significant reduction in response to both doses of fructose treatment (8.75 mM: 0.6-fold, *p* = 0.0005; 12.5 mM: 0.5-fold, *p* = 0.0002; Figure 2a). Similarly, MAP2 (70 kDa), which is expressed specifically during the embryonic period, showed a significant reduction in response to fructose treatment (8.75 mM: 0.8-fold, *p* = 0.0006; 12.5 mM: 0.7-fold, *p* = 0.0002; Figure 2b). Furthermore, GFAP protein expression showed a significant increase at both doses of fructose as compared to the control (8.75 mM: 1.3-fold, *p* = 0.001; 12.5 mM: 1.2-fold, *p* = 0.0002; Figure 2c).

### 2.2. Fructose Decreases Neurite Length with No Impact on the Number of Neurites

**Figure 3:**
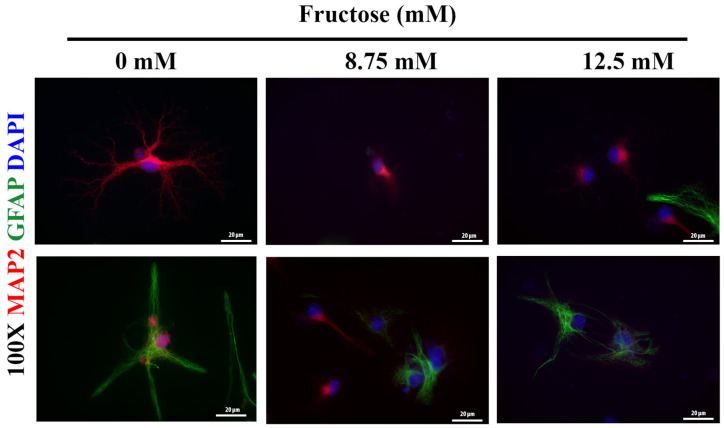
Effects of neuron and astrocyte morphology following fructose treatments. NSCs were differentiated into neurons and astrocytes for seven days. Following differentiation, an immunofluorescent staining of MAP2 (neurons in red) and GFAP (astrocytes in green) was performed. Images above at 100× magnification were captured with the Keyence BZ-X1000 microscope and neurite length, neurite count, the neuronal soma area, and neuronal soma perimeter were determined. Scale bar = 20 μm.

**Figure 4:**
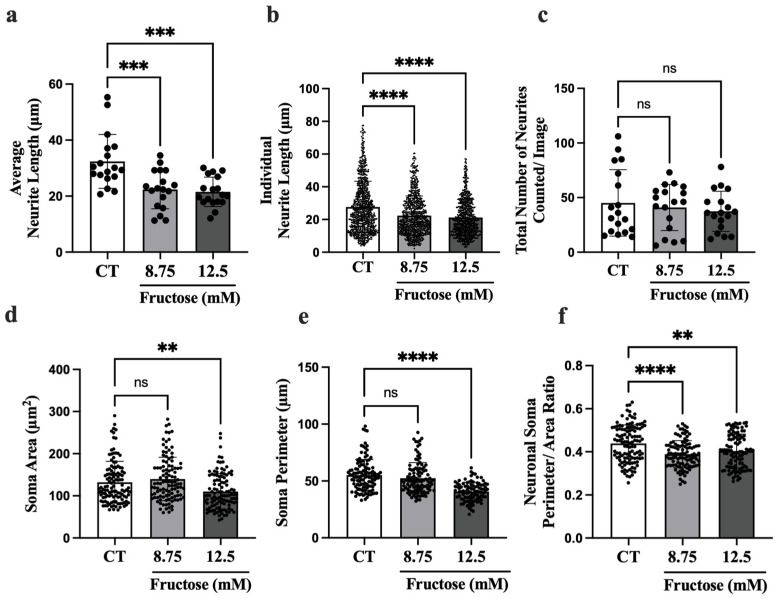
Fructose impairs neuronal morphogenesis and differentiation: Quantitative assessment. NSCs were treated with two doses (8.75, 12.5 mM) of fructose in differentiation medium for seven days. (**a**) Average and (**b**) individual neurite lengths, (**c**) total number of neurites per image, (**d**) soma area, (**e**) soma perimeter, and (**f**) soma perimeter-to-area ratio were analyzed. FIJI software (v1.54p.) was utilized for neuritic morphology analysis from images acquired at 40× magnification. ** *p* = 0.0019, *** *p* = 0.0002, **** *p* = 0.0001, ns = not significant.

To assess neuronal variability, the individual neurite lengths were measured. The neurite length was defined as the distance from the soma to the distal end of the projection. In response to low- and high-concentration fructose treatments, individual neurite lengths were significantly (*p* = 0.0001) shorter as compared to controls (8.75 mM: 23.8 ± 0.5 μm; 12.5 mM: 22.5 ± 0.5 μm; control: 29.9 ± 0.7 μm; Figure 3 and Figure 4a).

To understand the overall trend of treatment effects, the average of all neurite lengths per image was calculated. For each image, the average neurite length was calculated as the sum of all individual neurites divided by the total number of neurites. Consistent with individual neurite length, the average neurite length per image was also significantly (*p* = 0.0002) reduced in fructose-treated cells as compared to control (8.75 mM: 22.4 ± 1.6 µm; 12.5 mM: 21.5 ± 1.2 µm; control: 32.4 ± 2.3 µm; Figure 4b). However, the number of neurites per image was not impacted by fructose treatment (8.75 mM: 40.9 ± 5.0 µm; 12.5 mM: 37.4 ± 4.3 µm; control: 45.1 ± 7.2 µm; Figure 4c).

### 2.3. Assessing the Neuronal Soma Area, Perimeter, and Perimeter-to-Area Ratio

To further understand the neuronal morphological changes as a result of fructose treatment, the soma area of individual neurons was quantified to assess metabolic capacity and cellular volume [25], and soma perimeter was measured to evaluate membrane smoothness/shape and progression through neuronal morphological changes [26]. Additionally, the neuronal soma perimeter-to-area ratio was investigated to determine the complexity and morphological changes during fructose treatment. The neuron’s soma area and perimeter were significantly decreased in response to the higher fructose (12.5 mM) concentration as compared to controls (area: 108.8 ± 4.1 µm^2^ vs. 131.4 ± 4.6 µm^2^, *p* = 0.003; perimeter: 40.6 ± 0.8 µm vs. 55.2 ± 1.3 µm, *p* = 0.0001; Figure 4d,e). Whereas the neuronal soma perimeter-to-area ratio significantly (*p* = 0.0001) decreased at both concentrations of fructose treatment as compared to control (8.75 mM: 0.39 ± 0.01; 12.5 mM: 0.40 ± 0.01; control 0.44 ± 0.01; Figure 4f).

## 3. Discussion

The present study demonstrates important effects of in vitro fructose on NSC differentiation. The key findings show that fructose (1) promotes astroglial versus neuronal lineage, and (2) decreases neuronal cell body size as well as neurite growth. These results suggest impaired neurodevelopment and are consistent with in vivo mouse studies demonstrating that a maternal Western diet, particularly one high in fructose, causes neurological dysfunction [23], neuroinflammation [27], abnormal hippocampal development [28], decreased hippocampal neural stem cells and newborn neurons [29]. Furthermore, human lactation studies demonstrate that consumption of high-fructose beverages results in elevated milk fructose levels for up to five hours following intake [30]. In addition, gas chromatography analyses showed that many common infant formulas contained an overestimation of sugar content relative to reported values [31]. Together, these studies highlight that impaired neurodevelopment may begin as early as the embryonic stage, and can be exacerbated postnatally by fructose levels in breastmilk and formulas.

Whilst glucose is the primary energy source for NSCs and is readily taken up via the GLUT1 transporter, and in developing neurons by the GLUT3 transporter, fructose uptake is facilitated by GLUT5 transporters, which are primarily expressed on glial cells [32,33]. Once in the brain, fructose is rapidly metabolized, bypassing essential regulatory steps and leading to unregulated fructose conversion that requires substantial adenosine triphosphate (ATP) consumption. Consequently, this results in the accelerated reduction in ATP levels exceeding the cell’s ability for replenishment, contributing to increased oxidative stress, reactive gliosis, and potential neuroinflammation [34]. This is particularly evident in hyperglycemic conditions in which peripheral blood sugar levels can reach up to 20 mM though in the brain, the concentration rises to approximately 5 mM [35]. These physiologically relevant blood sugar levels served as a guide for selecting our experimental glucose and fructose concentrations, which we assessed at various combinations to elucidate the overall effects of fructose. Additionally, studies report that fructose concentration in umbilical cord blood is higher than in maternal blood, suggesting endogenous fructose production by the fetal–placental unit. Further, fructose concentrations are higher in newborns at 48 h after birth than in the fetal umbilical cord blood, suggesting that fructose production is a continuous process from fetus to newborn [36]. In a fetal pig, fetal blood fructose is 2.3 times higher than glucose levels [37].

The equimolar amount of glucose and fructose in Treatment 1 mimics a model of dietary exposure to both hexose sugars (ingested as sucrose) and their combined effects on neural development, whereas Treatment 2 reflects fructose as the predominant energy source (12.5 mM fructose) and is relevant for elucidating its effect on neurodevelopment impairment associated with excessive fructose intake [38,39].

The comparable effects seen with Treatments 1 and 2 may be attributed to attaining a threshold and saturation of specific enzymes and the transporter and metabolic pathways involved. Alternatively, the combined presence of glucose and fructose (Treatment 1), albeit at a lower dose, induces equivalent effects as that of a high fructose dose [34,40,41]. Nonetheless, studies suggest that fructose often promotes a greater state of cellular stress as compared to glucose [34,41]. Based on this, we speculate that the effects are primarily driven by fructose since glucose concentration in Treatment 1 was lower than that of Control.

Fructose-mediated preferential differentiation of NSCs towards astrogenesis versus neurogenesis was evident by both immunofluorescent imaging and Western blot analysis. The NSC differentiation is tightly and temporally regulated, with neurogenesis occurring prior to astrogenesis [42]. This transition is governed by a combination of intrinsic signaling molecules, transcription factors, epigenetic changes and environmental cues [43,44]. For induction of astrogenesis, binding of transcription factor STAT3 (signal transducer and activator of transcription) to GFAP is crucial. During the neurogenic phase, Dnmt1 maintains methylation of the GFAP promoter, preventing STAT3 access to GFAP and thereby suppressing astrocyte differentiation and promoting neurogenesis [45]. It is plausible that fructose treatment selectively inhibits neuronal differentiation or survival [46] rather than directly promoting astrogenesis. Future studies will investigate lineage-specific effects using lineage tracing, live-cell time-lapse microscopy, and single-cell RNA sequencing (scRNA-seq) to characterize changes in individual cell populations and determine the cellular mechanisms underlying this observed phenotype. The current study was undertaken to investigate the specific effects of fructose on the biological processes of hippocampal neural stem cells without confounding factors from surrounding organs or immune systems. As an initial step, we have demonstrated the changes in phenotypic characteristics due to in vitro fructose treatment, which now paves the way for the next series of studies to identify the underlying mechanisms.

The underlying mechanism of fructose-mediated altered neurogenesis may involve neuroinflammation, mitochondrial dysfunction, production of reactive oxygen species (ROS) and/or perturbed insulin signaling [38,40,47]. Specifically, fructose increases production of proinflammatory cytokines (TNF-α, IL-6, MIP-2) [47]. We have previously shown that TNFα and ROS treatment impair hypothalamic NSC mitochondrial function with increased production of ROS and decreased protein expression of pro-neurogenic transcription factors, Mash1/Ngn3. Notably, inhibition of STAT3 and NFκB prevented these effects [48]. It is plausible that the fructose-mediated inflammation reduces Dnmt1 and/or activates STAT3 and contributes to the decreased neuronal-to-astrocyte ratio. Studies show that deletion/inhibition of Dnmt1 activity and activation of STAT3 in NSCs result in precocious astroglia differentiation [49,50]. Notably, proinflammatory cytokines, such as TNFα, can activate STAT3 [51,52]. This is consistent with in vivo studies showing increased hippocampal inflammation and oxidative stress [53], as well as a reduction in neurogenesis in the subgranular zone (SGZ) of the dentate gyrus in adult mice consuming high fructose [39]. The reduced neurogenesis was further validated by a decline in long-term potentiation, which is due to the lack of immature excitatory neurons, suggesting that fructose alters the neuronal plasma membrane, leading to impaired neuronal excitability and signaling [54].

In the present study, fructose treatment altered neuronal morphological features, as evident by a significant decrease in neurite length and soma. Specifically, the reduction in both the individual (assesses neuronal variability) and average (reflects treatment effects) neurite length suggests decreased neuronal growth, maturity, and/or structural integrity, suggesting impaired neuronal extensions with simplified neuronal architecture.

Individual neurite lengths demonstrate the variation in projection range length across neurons, displaying the full distribution of neurite outgrowth, highlighting subtle differences in branching and the presence of shorter/longer extensions. In contrast, the average neurite length provides a cumulative measure of overall outgrowth and comparison of treatment-related trends. The reduced soma area and perimeter reinforce a compromised neuron with reduced metabolic capacity [55] and decreased axonal growth [56] as a result of fructose exposure. Although reduced soma is a hallmark feature of immature neurons, it can occur under cellular stress (i.e., increased inflammation, oxidative reactive species) [57,58] and is evident in certain neurodevelopmental disorders [59,60,61]. Additionally, the significant reduction in neuronal soma perimeter-to-area ratio suggests that the soma is more spherical and less complex. Thus, at higher fructose concentrations, neurons are adversely impacted with shorter neurites and smaller somas, signifying marked structural damage [62] with delayed progression in neuronal morphogenesis and differentiation [63]. Based on our findings, we speculate that fructose results in neurons with phenotypic characteristics of shorter immature neurites. Collectively, the data emphasize the importance of maintaining a balanced neuron–astrocyte ratio, as disruptions can alter tripartite synapse formation, ultimately contributing to impaired memory formation and susceptibility to neurodegeneration [64,65].

These findings are consistent with human and animal studies in which fructose induces hippocampal structural changes, as well as cytoskeletal and axonal growth inhibition [63]. Human studies demonstrate that children between the ages of 7 and 11 years who consumed excess fructose resulted in hippocampal inflammation in regions CA2/3 subfields and reduced synaptic pruning as evident by Magnetic Resonance Imaging [66]. Animal studies showed that rats consuming a high-fat and fructose diet for 7 days exhibited reduced hippocampal dendritic spine number and astrocyte activation [67]. Furthermore, a transcriptome analysis of the effects of a maternal fructose diet during gestation and lactation reported altered offspring hippocampal developmental programming [63].

The mechanism for fructose-mediated reduced neuronal growth/maturity may be driven by neuroinflammation, metabolic reprogramming, oxidative stress, inhibition of key insulin signaling molecules, mitochondrial dysfunction and/or reduced DNMT1 [39,67,68,69,70].

In conclusion, in vitro fructose treatment of hippocampal NSCs promotes astroglial versus neuronal lineage and compromises neuronal growth. Considering this, it is imperative to interrogate the mechanisms that shift NSC differentiation to astrocytes and impair neuronal structure, as this may lead to secondary effects on the foundation of neuronal synaptic connections, networks and function.

## 4. Materials and Methods

### 4.1. Materials and Reagents

Supplementary Table S1 summarizes the source of materials and reagents.

### 4.2. Neurosphere Culturing

C57BL/6 embryonic mouse NSCs (MUBNF-01001, Cyagen OriCell, Guangzhou, China) were cultured, harvested, and seeded according to the manufacturer’s protocol (MUBNF-01001). Briefly, NSCs were cultured in OriCell Neural Stem Cell Growth Medium with 1% antibiotic–antimycotic for two to three days, during which time they aggregated to form neurospheres. After which, neurospheres were harvested, dissociated into single cells using TrypLE Express Enzyme, and seeded in a proliferation medium (Gibco Neurobasal Media Plus and B-27 Plus Supplement, Waltham, MA, USA) for 24 h and thereafter prepared for differentiation and fructose treatment. Henceforth, these cells are referred to as neural stem cells (NSCs).

For differentiation, the media was replaced by basal differentiation medium (BDM), which consisted of glucose-free Dulbecco’s Modified Eagle Medium (DMEM) and diluted Ham’s F-12 Nutrient Mix. The control cells had a final concentration of 17.5 mM glucose (no fructose) and represent a physiological condition without exposure to high-fructose corn syrup (HFCS). For fructose treatment, cells were treated with 8.75 mM (Treatment 1) and 12.5 mM (Treatment 2) D-fructose and adjusted Ham’s F-12 Nutrient Mix to provide a final overall concentration of 17.5 mM hexose sugar. This was to ensure equivalent osmotic concentration for all groups (Table 1), and viability of cells as studies have shown that replacing glucose entirely with fructose triggers cellular stress, cytokine production and reduced proliferation [38]. Cells were treated with fresh medium every 48 h for 7 days and cells were thereafter processed for immunostaining or extracted for protein.

In Treatment 1, equal amounts (8.75 mM) of glucose and fructose were used to mimic a Western diet in which both hexose sugars are present. Treatment 2 reflected a high fructose concentration (12.5 mM). At these concentrations, there is negligible cell cytotoxicity as shown by previous studies [71,72]. All groups, including Control, provided isocaloric and osmolality consistency.

**Table 1:** Experimental Groups.

Experimental Groups	D-Glucose Concentration	D-Fructose Concentration	Final Sugar Concentration
Control (CTR)	17.5 mM	0 mM	17.5 mM
Treatment 1 (TRT1)	8.75 mM	8.75 mM	17.5 mM
Treatment 2 (TRT2)	5 mM	12.5 mM	17.5 mM

Control cells had a final concentration of 17.5 mM glucose (no fructose) and represent a physiological condition without exposure to high-fructose corn syrup (HFCS). In Treatment 1, equal amounts (8.75 mM) of glucose and fructose were used to mimic a Western diet in which both hexose sugars are present. Treatment 2 reflected a high fructose concentration (12.5 mM). At these concentrations, there is negligible cell cytotoxicity as shown by previous studies [72,73]. All groups, including Control, provided isocaloric and osmolality consistency.

### 4.3. Immunofluorescent Staining

Cells were rinsed with TBS/BSA solution (Tris-Buffered Saline, 1% bovine serum albumin), fixed in 4% paraformaldehyde, and subsequently washed with 0.1% triton in TBS (TBST). Following this, non-specific antibody binding was blocked with 10% donkey serum containing 2% BSA and TBST for one hour at room temperature. Primary antibodies (neuronal cell body and dendrite marker MAP2 and astrocyte marker GFAP) were added and incubated overnight at 4 °C and subsequently exposed to secondary antibodies (donkey anti-rabbit IgG, Alexa Fluor 594, and donkey anti-mouse IgG, Alexa Fluor 488) for one hour at room temperature. Cells were washed with TBST, followed by a nuclear stain with DAPI (1 µg/mL). *n* = 3 independent cultures were used.

### 4.4. Neuronal and Astrocyte Counts

For cell count study, only a higher dose of fructose (12.5 mM) was used. From each culture and treatment, twelve immunostained images within similar regions of the well were captured using the Keyence BZ-X800 microscope (10× magnification, Osaka, Japan). Positive neuronal cells were identified by a NeuN, MAP2, and DAPI co-localized positive stain, and astrocyte cell identified by GFAP- and DAPI-positive co-localized staining. We calculated an approximately 285–300 total number of DAPI-positive cells per image, using 12 images per replicate, analyzing three independent replicates for each experimental condition. The positive stained neuronal and astrocyte cell count was determined utilizing a manual cell counter on FIJI software. Results are presented as a percentage of the total number of cells. Although image analysis was non-blinded, we minimized observer bias by using identical imaging conditions and standardized FIJI parameters for all experimental groups.

### 4.5. Neuronal Morphological Analysis

**Figure 5:**
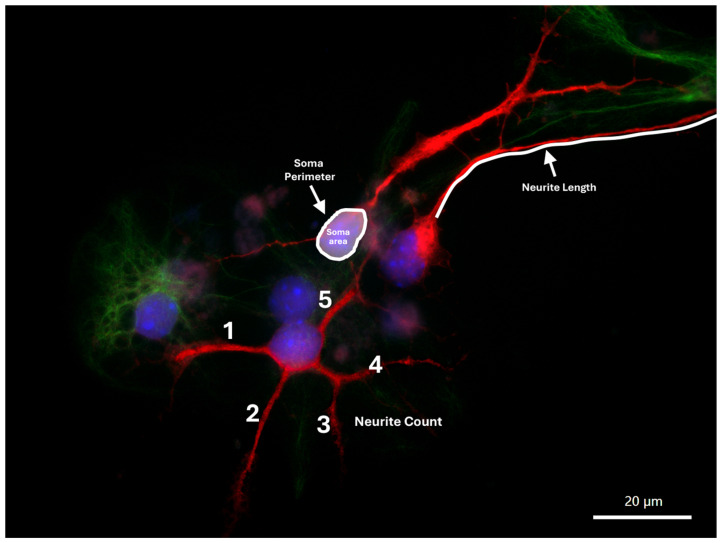
Quantification of neuronal morphology. Neurons were identified by an immunofluorescent staining of MAP2 (red), astrocytes by GFAP (green) and nuclei by DAPI (blue). Images were taken at a 40× magnification with the Keyence BZ-X800 microscope. FIJI software was utilized to determine neurite length, neurite count, the neuronal soma area, and neuronal soma perimeter.

Quantitative analysis of neuronal dendrites/axons (neurites) length and number, including neuronal cell body size (soma) and perimeter, was determined using FIJI software. For neurite measurements, MAP2-targeted images (40× magnification) were captured within similar regions of the well per treatment using the Keyence BZ-X800 Microscope. For each MAP2-stained neuron image, all individual neurite lengths (i.e., single projections from a neuron) were measured to assess cellular variability. Average neurite length per image is also reported to reflect treatment effects. Additionally, the total number of neurites per MAP2-stained neuron image was counted (Figure 5). To reflect neuronal metabolic changes and membrane shape, neuronal soma area and perimeter were calculated by drawing the outer edge of the cell body and measuring the corresponding MAP2-positive area (i.e., excluding any neurite extension length) on IF images. To understand neuronal soma morphology and complexity, the ratio of the soma perimeter to area was determined.

### 4.6. Protein Extraction

Protein was extracted, and expression was determined as previously described by us [27]. Briefly, the cells were harvested, dissolved in RIPA solution with a protease inhibitor cocktail, and sonicated, and the cell lysates were processed for quantification of protein concentration using Pierce™ BCA Protein Assay Kits (Waltham, MA, USA) following the protocol as described by the manufacturer.

### 4.7. Western Blot

Westerns were performed as previously reported by our group [45]. Briefly, 25 μg of protein samples were loaded and separated on a 15% SDS-PAGE gel and transferred to a nitrocellulose membrane. After blocking in 5% BSA in tris-buffered saline with TBST, the membrane was incubated overnight at 4 °C in primary antibodies MAP2 (1:1000) and GFAP (1:5000). Thereafter, the membranes were washed in TBST and incubated with a secondary antibody for one hour at room temperature. Protein band visualization was performed with the Bio-Rad ChemiDoc MP Imaging System (Hercules, CA, USA). The total protein was used as the loading control, and MAP2 and GFAP were normalized to the loading protein.

### 4.8. Statistical Analysis

N = 3 independent cultures were undertaken. GraphPad Prism (Version 10.6.0 (796)) was utilized for data analysis. For IF neuronal morphology analysis, the ROUT test was implemented to find outliers in the data set. For neuronal and astrocyte counts, the unpaired *t*-test was used. For neuronal morphology and protein expression data, a one-way ANOVA test followed by a post hoc Bonferroni test was performed. The data of *n* = 3 independent experiments are presented as mean ± standard error of mean (SEM) with statistical significance set at *p* < 0.05. For Western blot analysis, values are normalized to untreated control cells and reported as fold change.

## 5. Conclusions

Fructose treatment reduced the neuron-to-astrocyte ratio, as evident by both immunofluorescence and Western blot analyses. Also, fructose impairs neuronal morphology by significantly reducing both average neurite length and individual neurite length at both fructose concentrations, while a higher concentration (12.5 mM) decreases neuronal soma area and perimeter. Furthermore, the neuronal soma perimeter-to-area ratio was significantly reduced at both fructose concentrations, suggesting that fructose impairs neuronal structural complexity. Future investigations will explore the mechanistic effects of fructose at a transcriptional level including the role of epigenetic factors (e.g., Dnmt1), mitochondrial bioenergetics (e.g., PGC-1α), and inflammatory and oxidative molecules, using single-cell RNA sequencing (scRNA-seq) in vitro and in vivo models. Collectively, these investigations may provide further insight into understanding the mechanistic effects of fructose during NSC differentiation and neuronal morphology. Understanding this may lay the groundwork for therapeutic approaches to mitigate the effects of fructose during neurodevelopmental programming.

## Data Availability

The original contributions presented in this study are included in the article/Supplementary Material. Further inquiries can be directed to the corresponding author.

## Supplementary Materials

The following supporting information can be downloaded at: https://www.mdpi.com/article/10.3390/ijms27156653/s1.

## Abbreviations

The following abbreviations are used in this manuscript:

ANOVA	Analysis of Variance
ATP	Adenosine triphosphate
BCA	Bicinchoninic acid
BDM	Basal differentiation medium
BSA	Bovine serum albumin
CA2/3	Cornu Ammonis 2 & 3
CTR	Control
DAPI	4′,6-diamidino-2-phenylindole dihydrochloride
DMEM	Dulbecco’s Modified Eagle Medium
DNMT1	DNA methyltransferase 1
GFAP	Anti-glial fibrillary acidic protein
GLUT1	Glucose Transporter Type 1
GLUT3	Glucose Transporter Type 3
GLUT5	Glucose Transporter Type 5
IF	Immunofluorescence
IgG	Immunoglobulin G
Il-6	Interleukin-6
FIJI	Fiji is just ImageJ (v1.54p.)
MAP2	Microtubule-associated protein 2
Mash1	Mammalian achaete-scute homolog 1
MIP-2	Macrophage inflammatory protein-2
NeuN	Neuronal nuclei antigen
NFκB	Nuclear factor kappa-light-chain-enhancer of activated B cells
Ngn	Neurogenin-3
NSC	Neural stem cell
PFK-1	Phosphofructokinase-1
PGC-1α	Peroxisome proliferator-activated receptor gamma coactivator 1-alpha
RIPA	Radio-immunoprecipitation assay
ROUT	Regression and outlier unification testing
ROS	Reactive oxygen species
SDS-PAGE	Sodium dodecyl sulfate–polyacrylamide gel electrophoresis
SEM	Standard error of the mean
SGZ	Subgranular zone
STAT3	Signal transducer and activator of transcription
TBS	Tris-buffered saline
TBST	Tris-buffered saline with Tween 20
Tcf7l2	Transcription Factor 7-Like 2
TNF-α	Tumor Necrosis Factor-alpha
TRT1	Treatment 1
TRT2	Treatment 2
Zbtb16	Zinc Finger and BTB Domain Containing 16
