# Supplementary material for: D-Fructose Exposure Impairs Neuronal Development in Mouse Neural Stem Cells
Source: Int J Mol Sci. 2026 Jul 25;27(15):6653. doi: 10.3390/ijms27156653 (PMC13465542; doi:10.3390/ijms27156653)
Supplement: Supplementary file 1 [file ijms-27-06653-s001.zip › ijms-4377964-supplementary.pdf]

**Supplementary Table S1. Summary of Experimental Reagents & Resources.**

| REAGENT or RESOURCE | SOURCE | IDENTIFIER |
|---|---|---|
| OriCell™ C57BL/6 Mouse Neural Stem Cells | Cyagen Biosciences (Guangzhou) Inc | MUBNF-01001 |
| OriCell™ Neural Stem Cell Growth Medium | Cyagen Biosciences (Guangzhou) Inc | GUXNX-90011 |
| Antibiotic-antimycotic | Gibco | 15240-062 |
| Neurobasal Media Plus | Gibco | A35829-01 |
| B-27 Plus Supplement (50X) | Gibco | A3582801 |
| Dulbecco's Modified Eagle Medium (DMEM), no glucose | Gibco | 11966-025 |
| Ham's F-12 Nutrient Mix (1X) | Gibco | 11765-054 |
| TrypLE™ Express Enzyme (1X), no phenol red | Gibco | 12604013 |
| Glucose (D-(+)-Glucose 299.5% (GC) | Sigma Aldrich | G8270 |
| D-(-)-fructose 299% | Sigma Aldrich | F3510 |
| T25 Nunc™ Non-treated Flasks | Thermo Scientific™ | 169900 |
| MAP2 | Invitrogen | PA1-10005 |
| GFAP | Sigma-Aldrich | G3893 |
| Donkey anti-rabbit IgG, Alexa Fluor 594 | Thermo Fisher Scientific | A21207 |
| Donkey anti-mouse IgG, Alexa Fluor 488 | Thermo Fisher Scientific | A21202 |
| DAPI (1mg/ml) | Invitrogen | 62224 |
| 6-well plate | TPP | 92006 |
| Cell scraper | Fisher Scientific | 50-101-128 |
| 2.0ml MCT Graduated natural tubes | Fisherbrand | 05-408-138 |
| 10X RIPA Buffer | Cell Signaling Technology | 9806S |
| Pierce™ BCA Protein Assay Kits | Thermo Scientific | 23227 |
| Criterion XT Precast Gel | Bio-Rad | 3450124 |
| Criterion Cell | Bio-Rad | Criterion Cell |
| Midi Format 0.2 μm Nitrocellulose | Bio-Rad | 1704159 |
| PowerPac | Bio-Rad | PowerPac |
| Trans-Blot Turbo Transfer System Cassette Base | Bio-Rad | Trans-Blot Turbo System |
| 1X MOPS | Bio-Rad | 1610788 |
| XT | Bio-Rad | 161-0363 |
| Ponceau S | Santa Cruz Biotechnology | sc-301558 |

| | | |
|---|---|---|
| BioRad Plus Protein Dual Color Standards | Bio-Rad | 1610374 |
| Goat Anti-Rabbit IgG (H + L)-HRP Conjugate | Bio-Rad | 1706515 |
| Goat Anti-Mouse IgG (H + L)-HRP Conjugate | Bio-Rad | 1706516 |
| SuperSignal™ West Pico PLUS Chemiluminescent Substrate | Thermo Fisher Scientific | A8403739 |
| 10x Tris Buffered Saline (TBS) | Bio-Rad | 1706435 |
| Bovine serum albumin (BSA) | Sigma-Aldrich | A7906 |
| Paraformaldehyde Solution 4% in PBS | ChemCruz | SC-281692 |
| Donkey Serum | Millipore | S30-M |
| Keyence BZ-X800 Microscope | Keyence | BZ-X800 |
| Keyence BZ-X1000 Microscope | Keyence | BZ-X1000 |
| ChemiDoc™MP Imaging System | Bio-Rad | ChemiDoc™MP |
